# Supplementary material for: The effects of genital myiasis on the diversity of the vaginal microbiota in female Bactrian camels
Source: BMC Vet Res. 2022 Mar 5;18:87. doi: 10.1186/s12917-022-03189-5 (PMC8897907; doi:10.1186/s12917-022-03189-5)
Supplement: Supplementary file 5 — Additional file 5. [file 12917_2022_3189_MOESM5_ESM.zip › MPL201709200_16s_yy/Treat1/B12_pca/3D-PCA.pdf]

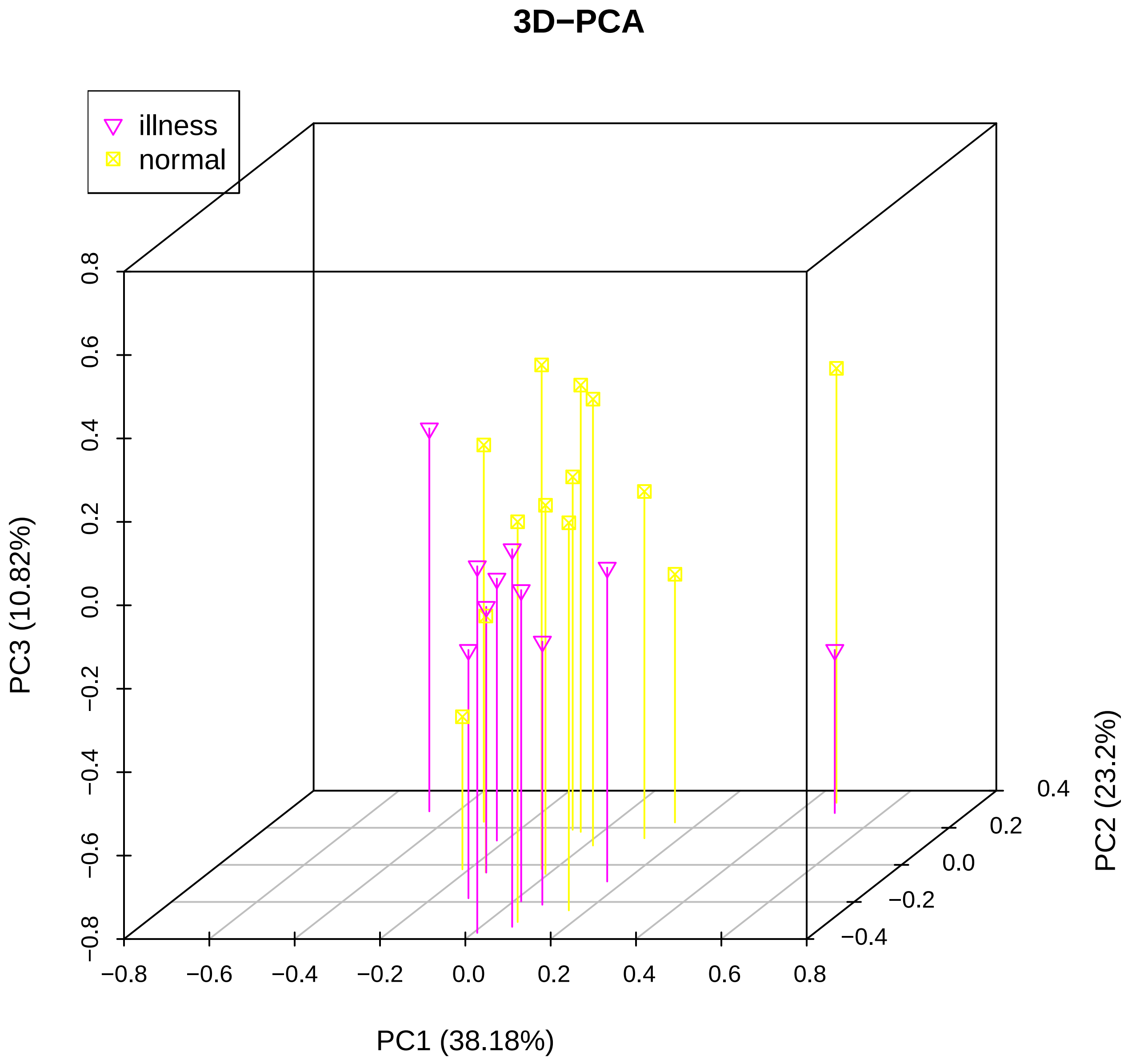
3D−PCA
illness
normal
PC1 (38.18%)
PC2 (23.2%)
PC3 (10.82%)
−0.8
−0.6
−0.4
−0.2
0.0
0.2
0.4
0.6
0.8
